# Prevalence of Chronic Cardiovascular and Metabolic Diseases in Senegalese Workers: A Cross-Sectional Study, 2010

**DOI:** 10.5888/pcd10.110339

**Published:** 2013-01-03

**Authors:** Sidy Mohamed Seck, Serigne Guéye, Kéba Tamba, Issa Ba

**Affiliations:** Author Affiliations: Serigne Guéye, University Hospital, Aristide Le Dantec, Dakar, Sénégal; Kéba Tamba, Laboratoires Roche, Dakar, Sénégal; Issa Ba, Regional University Hospital, Saint-Louis, Sénégal.

## Abstract

**Introduction:**

Noncommunicable diseases (NCDs) are a major public health threat, particularly in developing countries. In sub-Saharan Africa, the scarcity of reliable data on NCDs in the general population makes it difficult to develop efficient prevention strategies. The objective of this cross-sectional study was to assess the prevalence of 4 cardiometabolic NCDs among 402 private-sector workers in Dakar, Senegal: high blood pressure (HBP), diabetes, obesity, and chronic kidney disease (CKD).

**Methods:**

We collected demographic, clinical, and biological data for each worker during routine occupational health visits between September 1 and November 30, 2010. Multivariate analyses were performed to identify risk factors associated with NCDs.

**Results:**

Among the 402 study participants, 24.1% had HBP, 9.7% had diabetes, 16.7% were obese, and 22.4% had CKD. About half of participants (48.5%) were not aware of their diseases before the screening. Univariate analysis showed that age was significantly associated with blood pressure, fasting blood glucose, and renal function. After adjusting for age and sex, systolic blood pressure was correlated with renal function, and physical inactivity was significantly associated with obesity.

**Conclusion:**

Despite its small sample size, our study provides a perspective on the extent of cardiometabolic NCDs in Senegalese workers. Our study also suggests that targeted screening activities focusing on socio-professional groups may be helpful in the absence of national integrated prevention programs.

## Introduction

Chronic noncommunicable diseases (NCDs) are the greatest world public health challenge of the 21st century (1,2). In 2008, approximately 36 million deaths were attributable to NCDs; most occurred in resources-limited countries, and more than half were due to diabetes and cardiovascular diseases (3). The social and economic burden of NCDs is great because they are associated with high rates of premature deaths in adults younger than 60 (2,4). In response to this epidemic of NCDs, the World Health Organization (WHO) recommends population-based screening and prevention strategies. In sub-Saharan Africa, reliable data on NCDs at the population level are lacking (5), and in many countries integrated national programs that provide such data have not been implemented (2). In Senegal, previous studies of urban areas reported prevalence of 10.4% for hypertension (6), 17.9% for diabetes (7), and 31.2% for overweight and obesity (2). Staff providing primary health care services are often not equipped with skills necessary to provide care for NCDs, and the few medical specialists in Senegal are concentrated in the capital, Dakar. For private workers, annual occupational health visits are mandatory; however, health care providers often do not check patients for NCDs. If a health care provider does routinely measure blood pressure and weight, other factors such as renal function and obesity are not assessed, even in high-risk people. This study’s objective was to assess the prevalence of 4 common cardiometabolic NCDs and their associated modifiable risk factors among private-sector workers in Dakar.

## Methods

We performed a population-based, cross-sectional study in 2 major private-sector information technology companies in Dakar. A representative sample of workers was randomly selected and screened during routine occupational health visits for high blood pressure (HBP), diabetes, obesity, and chronic kidney disease (CKD). The inclusion criteria for the study were being an employee of 1 of the 2 companies and willingness to participate. Participation in the study was free, and we obtained informed consent from each participant. We used a questionnaire to collect demographic data. Those who did not want to undertake the questionnaire were excluded from the study. We estimated socioeconomic status of participants on the basis of their educational level and total self-reported annual income. In addition to demographic data, for each participant, we measured blood pressure (BP), weight, and height; assessed physical activity level and tobacco and alcohol use; and measured fasting blood glucose (FBG), serum creatinine, and urinary albumin excretion (UAE). Skilled doctors and nurses performed all measurements. To assess tobacco and alcohol use, we asked participants if they currently smoked, had ever smoked, and if they had formerly smoked, had they stopped for a year or more. For alcohol, we asked how many drinks the person consumed in a week. Anyone consuming more than 1 drink per week was classified as a drinker. We calculated body mass index (BMI) as weight in kilograms divided by height in meters squared (kg/m^2^). Participants were identified as having hypertension, according to the Seventh Report of the Joint National Committee on Prevention, Detection, Evaluation, and Treatment of High Blood Pressure standards, if they had a BP at or higher than 140/90 mm Hg or had taken antihypertensive drugs in the previous 2 weeks (8). Participants with FBG at or higher than 1.26 g/L or who were on antidiabetic drug therapy were described as having diabetes. Participants with a BMI of 30 or higher were considered obese (9). Glomerular filtration rate (GFR) was estimated by using the Modification of Diet in Renal Disease (MDRD) equation, and UAE was measured with urine strips. CKD was defined according to National Kidney Foundation guidelines (estimated GFR <60 mL/min or presence of albuminuria detected in 1 morning urine sample using Combur-Test (Roche diagnostics, Mannheim, Germany) (10). We assessed participants’ awareness of a diagnosis of an NCD by asking if they had been told by a health professional during the previous 12 months that they had the disease or if they were undergoing a pharmacological or nonpharmacological treatment of the disease. Physical inactivity was defined as less than 30 minutes per day of moderate-intensity aerobic activity or less than 20 minutes per day of vigorous-intensity aerobic physical activity or the equivalent (11). Those with abnormal laboratory values were referred to a physician for further examination and treatment. Statistical analyses were performed with SPSS 16.0 (IBM Corporation, Chicago, Illinois). Continuous variables were presented as mean (standard deviation), and categorical variables were expressed as percentage. We used χ^2^ test and Student *t* test, as appropriate, to compare proportion and means. We used multiple regression analysis to assess the likelihood of different NCDs according to demographic and clinical data. *P* ≤ .05 was considered significant.

## Results

From a target population of 756 workers (502 men and 254 women), we enrolled 402 (53.2%) participants in the study, and all of them completed the screening. All participants had received some formal education, and 86.8% had completed secondary or higher education. Only 15.4% of participants were of low socioeconomic level; all others had annual incomes over the national average. Prevalence of HBP, diabetes, obesity, and CKD in the total population was 24.1%, 9.7%, 16.7%, and 22.4%, respectively (Table 1). Thirty-two percent of participants with hypertension, 23.0% with diabetes, 17.6% who were obese, and 89.0% with CKD were not aware of their diseases before the screening.

**Table 1:** Demographic and Clinical Characteristics of Participants (N = 402), Study of Prevalence of Noncommunicable Diseases (NCDs) in Senegalese Workers, 2010^a^

Characteristic	All Participants (N = 402)	Men (n = 266)	Women (n = 136)	*P* Value
**Demographic**				
Age, mean (SD), y	46.2 (7.6)	46.9 (7.7)	44.7 (7.3)	.01
Personal history of NCDs	81 (20.1)	49 (18.4)	32 (23.5)	.14
Family history of NCDs	165 (41.0)	89 (33.4)	76 (55.9)	.82
Tobacco use	28 (7)	20 (7.5)	8 (5.9)	.54
Alcohol use	54 (13.5)	48 (18.0)	6 (4.4)	.001
Physical inactivity	174 (43.3)	106 (39.8)	68 (50.0)	.05
**Clinical**				
Systolic BP, mm Hg	131.0 (22.0)	131.3 (21.0)	130.5 (21.0)	.81
Diastolic BP, mm Hg	82.9 (12.7)	82.8 (13.0)	83.0 (12.2)	.71
Hypertension stage 1^b^	49 (12.1)	32 (12.0)	18 (13.2)	.72
Hypertension stage 2^c^	48 (11.9)	38 (14.3)	10 (7.3)	.70
BMI, kg/m^2^	25.9 (4.3)	25.3 (4.0)	27.1 (4.7)	.002
<25	9 (2.2)	8 (3.0)	1 (0.7)	.005
≥25 to <30	323 (81.0)	221 (55)	102 (75)	.01
≥30	67 (16.7)	34 (12.8)	33 (24.2)	.48
FBG, g/L	1.04 (0.3)	1.06 (0.32)	1.01 (0.96)	.11
<1.10	319 (79.6)	201 (75.5)	118 (86.7)	.44
1.11 to <1.25	43 (10.7)	33 (12.8)	10 (7.3)	.29
≥1.26	39 (9.7)	31 (11.6)	8 (5.8)	.43
Serum creatinine, mg/dL	1.1 (0.3)	0.9 (0.4)	1.0 (0.3)	.60
eGFR, ml/min^d^	99.8 (23.5)	99.6 (24.8)	97.8 (22.4)	.90
Albuminuria >1g/L	22 (5.5)	15 (5.6)	07 (5.1)	.42
CKD stage 1^e^	50 (12.4)	34 (12.8)	16 (11.7)	.21
CKD stage 2^f^	32 (8.2)	20 (5.7)	12 (8.8)	.18
CKD stage 3^g^	8 (2.0)	5 (0.3)	3 (2.2)	.53

Abbreviations: SD, standard deviation; BP, blood pressure; BMI, body mass index; FBG, fasting blood glucose; eGFR, estimated glomerular filtration rate; CKD, chronic kidney disease.

a All values are expressed as n (%), unless otherwise indicated.

b BP between 140/90 mm Hg and 159/99 mm Hg.

c BP ≥160/100 mm Hg.

d Estimated glomerular filtration rate according to Modification of Diet in Renal Disease equation.

e CKD stage 1 is an eGFR ≥90 ml/min, or the presence of albuminuria, or both.

f CKD stage 2 is an eGFR 60–89 mL/min, or the presence of albuminuria, or both.

g CKD stage 3 is an eGFR 30–59 mL/min, or the presence of albuminuria, or both.

More than half of participants with a diagnosed NCD had 2 diseases or more (Table 2). This comorbidity was most remarkable in participants with diabetes, who all had an associated NCD.

**Table 2:** Distribution of Noncommunicable Diseases (NCDs) and Association of Diseases in Participants With Detected NCDs, Study of Prevalence of Chronic Cardiovascular and Metabolic Diseases in Senegalese Workers (N = 402), 2010

NCD	Participants with NCDs, n (%)	All participants, %
**Participants with 1 NCD**		
Obesity	32 (17.8)	8.0
CKD	28 (15.5)	7.0
HBP	28 (15.5)	7.0
Diabetes	0	0.0
**Participants with 2 NCDs**		
HBP and CKD	40 (22.2)	10.0
Diabetes and obesity	18 (10.0)	4.5
Diabetes and CKD	5 (2.8)	1.2
Diabetes and HBP	5 (2.8)	1.2
HBP and obesity	2 (1.1)	0.5
**Participants with ≥3 NCDs**		
HBP and obesity and CKD	11 (6.1)	2.7
HBP and diabetes and CKD	7 (3.9)	1.7
HBP and diabetes and obesity	4 (2.2)	1.0
**Total**	**180 (100)**	**44.7**

Abbreviations: CKD, chronic kidney disease; HBP, high blood pressure.

According to univariate analyses, as age increased, so did blood pressure (odds ratio [OR], 2.33; *P* = .04), FBG (OR, 1.14; *P* = .05), and eGFR (OR, 0.56; *P* < .01); physical inactivity was associated with male sex (OR, 0.67; *P* = .03). Socioeconomic status, education level, and sex were not correlated with BP, FBG, BMI, or eGFR. After multivariate regression analysis adjusting for age and sex, only SBP was correlated with an eGFR lower than 60 mL/min/1.73 m^2^ (OR, 2.1; *P* = .02), and physical inactivity was associated with a 40% increased risk for obesity (OR, 1.4; *P* = .01).

## Discussion

Data on the epidemiology of NCDs in sub-Saharan Africa are scarce and mainly represented by reports from South Africa. Few reports came from west Africa (5,12). Our study shows high prevalence of NCDs in Senegalese private-sector workers. Hypertension was the most frequent NCD observed in our study. Reported prevalence in sub-Saharan Africa is between 6% and 48% (5,13,14) with a large rural-urban disparity and large increase with age (12,15,16). Early studies found lower prevalences, but this difference can be explained by the higher cutoff used to define HBP (17). High-income countries are still the most affected by the epidemic of NCDs, but the number of people with hypertension is expected to increase during the next few years by 80% in developing countries and by 24% in the developed world (18).

Unexpectedly, CKD (low eGFR or albuminuria) was, after HBP, the most frequent NCD in our population. Population-based studies showed lower prevalence in adults living in the Congo (12.4%) and Ghana (13.2%) (19,20). These findings are alarming because low eGFR is a predictor of cardiovascular events and all-cause mortality (21), and even low-level albuminuria (1+) increases risk for cardiovascular disease by 30% (22).

Obesity in our population did not reach proportions observed in developed countries (2), but its prevalence was consistent with previously reported data in sub-Saharan African adults (5,13,23,24). However, obesity progresses faster in developing countries, and prevalence has doubled over the last 3 decades (25). The high prevalence of obesity and overweight could be related to higher frequency of physical inactivity among Senegalese workers compared with data for workers in low-income countries (18% in men and 21% in women) (2).

Diabetes prevalence in our study group was similar to the global prevalence, which in 2008 was estimated to be 9.8% for men and 9.2% for women (26). In sub-Saharan Africa, reported prevalence from community-based studies ranges from 0% to 16.0% with marked rural-urban differences (5,27). A recent screening of adults living in Dakar showed that 17.9% of participants had FBG at or higher than 1.10 g/L (7).

The high prevalence of NCDs among participants in our study contrasts with their low rate of disease awareness despite relatively high educational levels. Ignorance of disease risk is commonly found in studies addressing NCDs (14,28,29).

Our study has several limitations. First, we restricted our sample size to a specific profession that may not be representative of the whole Senegalese population. Second, the ranges for BMI we used in our study may not be appropriate for African populations and may need to be lowered, just as they were lowered for Asians (30). Third, measuring UAE in only 1 urine specimen could result in false-positive and false-negative results (31), and estimation of GFR in black Africans using the MDRD equation may overestimate renal function, particularly in people older than 60 years (20).

Evidence demonstrates that globalization of lifestyle and dietary habits is contributing to an epidemiological transition in middle and low-income countries (2). Concomitantly with infectious diseases, NCDs are growing rapidly while health care resources remain scarce (4). WHO recommends early detection and prevention of NCD risk factors in high-risk populations as cornerstone strategies for dealing with this new paradigm (2,3). However, identification of high-risk individuals might be difficult in sub-Saharan Africa where most of the population does not consult a health care professional for preventive care because of the shortage and inaccessibility of health care resources (32,33). In this context, any visit to a health care professional should be treated as an opportunity to assess individual risk for NCDs. HIV prevention and management programs in many African countries have developed strong health infrastructures and human resources that could be used for NCD programs. Compared with the majority of unemployed people in Senegal, many socio-professional groups are more able to afford regular medical check-ups because they have health coverage. Also, for private companies employing Senegalese workers, such preventive health strategies would be cost-effective inasmuch as one of the most significant socioeconomic effects of NCDs is the high financial cost of health insurance and loss of productivity (4).

Despite these limitations, our study provides a perspective on the burden of cardiovascular and metabolic diseases in a segment of the Senegalese population. Hypertension and CKD are the most frequent NCDs in this population, and awareness is low. Opportunistic screening for common preventable risk factors could improve early detection and management of NCDs in resource-limited countries like Senegal.

## References

[1] Anderson GF , Chu E . Expanding priorities: confronting chronic disease in countries with low income. N Engl J Med 2007;356(3):209–11. 10.1056/NEJMp068182 17229946

[2] Alwan A , Armstrong T , Cowan M , Riley L . Noncommunicable Diseases Country Profiles 2011. Geneva (CH): World Health Organization; 2011.

[3] Alwan A , Maclean DR , Riley LM , d’Espaignet E , Mathers CD , Stevens GA , Monitoring and surveillance of chronic noncommunicable diseases: progress and capacity in high-burden countries. Lancet 2010;376(9755):1861–8. 10.1016/S0140-6736(10)61853-3 21074258

[4] Abegunde DO , Mathers CD , Adam T , Ortegon M , Strong K . The burden and costs of chronic diseases in low-income and middle-income countries. Lancet 2007;370(9603):1929–38. 10.1016/S0140-6736(07)61696-1 18063029

[5] Dalal S , Beunza JJ , Volmink J , Adebamowo C , Bajunirwe F , Njelekela M , Non-communicable diseases in sub-Saharan Africa: what we know now. Int J Epidemiol 2011;40(4):885–901. 10.1093/ije/dyr050 21527446

[6] Astagneau P , Lang T , Delarocque E , Jeannee E , Salem G . Arterial hypertension in urban Africa: an epidemiological study on a representative sample of Dakar inhabitants in Senegal. J Hypertens 1992;10(9):1095–101. 1328370

[7] Duboz P , Chapuis-Lucciani N , Boëtsch G , Gueye L . Prevalence of diabetes and associated risk factors in a Senegalese urban (Dakar) population. Diabetes Metab 2012;38(4):332–6. 10.1016/j.diabet.2012.02.011 22521041

[8] Chobanian AV , Bakris GL , Black HR , Cushman WC , Green LA , Izzo JL Jr , The Seventh Report of the Joint National Committee on Prevention, Detection, Evaluation, and Treatment of High Blood Pressure: the JNC 7 report. Hypertension 2003;42(6):1206–52. 10.1161/01.HYP.0000107251.49515.c2 14656957

[9] The IDF consensus worldwide definition of the metabolic syndrome. Brussels (BE):International Diabetes Foundation; 2005. http://www.idf.org/metabolic-syndrome.

[10] Levey AS , Coresh J , Balk E , Kausz AT , Levin A , Steffes MW , National Kidney Foundation practice guidelines for chronic kidney disease: evaluation, classification, and stratification. Ann Intern Med 2003;139(2):137–47. 12859163 10.7326/0003-4819-139-2-200307150-00013

[11] Haskell WL , Lee IM , Pate RR , Powell KE , Blair SN , Franklin BA , Physical activity and public health: updated recommendations for adults from the American College of Sports Medicine and the American Heart Association. Circulation 2007;116(9):1081–93.. 10.1161/CIRCULATIONAHA.107.185649 17671237

[12] Addo J , Smeeth L , Leon DA . Hypertension in sub-Saharan Africa: a systematic review. Hypertension 2007;50(6):1012–8. 10.1161/HYPERTENSIONAHA.107.093336 17954720

[13] Sodjinou R , Agueh V , Fayomi R , Delisle H . Obesity and cardio-metabolic risk factors in urban adults of Benin: relationship with socio-economic status, urbanisation, and lifestyle patterns. BMC Public Health 2008;8:84. 10.1186/1471-2458-8-84 18318907 PMC2315643

[14] Macia E , Duboz P , Gueye L . Prevalence, awareness, treatment and control of hypertension among adults 50 years and older in Dakar, Senegal. Cardiovasc J Afr 2012;23(5):265–9. 10.5830/CVJA-2011-039 22002461 PMC3721830

[15] Bovet P , Ross AG , Gervasoni JP , Mkamba M , Mtasiwa DM , Lengeler C , Distribution of blood pressure, body mass index and smoking habits in the urban population of Dar es Salaam, Tanzania, and associations with socioeconomic status. Int J Epidemiol 2002;31(1):240–7. 10.1093/ije/31.1.240 11914327

[16] Agyemang C . Rural and urban differences in blood pressure and hypertension in Ghana, West Africa. Public Health 2006;120(6):525–33. 10.1016/j.puhe.2006.02.002 16684547

[17] Mufunda J , Chatora R , Ndambakuwa Y , Nyarango P , Kosia A , Chifamba J , Emerging non-communicable disease epidemic in Africa: preventive measures from the WHO regional office for Africa. Ethn Dis 2006;16(2):521–6. 17682258

[18] Kearney PM , Whelton M , Reynolds K , Munter P , Whelton PK , He J . Global burden of hypertension: analysis of worldwide data. Lancet 2005;365(9455):217–23. 15652604 10.1016/S0140-6736(05)17741-1

[19] Sumaili EK , Krzesinski JM , Cohen EP , Nseka NM . Epidemiology of chronic kidney disease in the Democratic Republic of Congo: review of cross-sectional studies from Kinshasa, the capital. Nephrol Ther 2010;6(4):232–9. 10.1016/j.nephro.2010.03.008 20409770

[20] Eastwood JB , Kerry SM , Plange-Rhule J , Micah FB , Antwi S , Boa FG , Assessment of GFR by four methods in adults in Ashanti, Ghana: the need for an eGFR equation for lean African populations. Nephrol Dial Transplant 2010;25(7):2178–87. 10.1093/ndt/gfp765 20100724 PMC2891745

[21] McCullough PA , Li S , Jurkovitz CT , Stevens LA , Wang C , Collins AJ , CKD and cardiovascular disease in screened high-risk volunteer and general populations: the Kidney Early Evaluation Program (KEEP) and National Health and Nutrition Examination Survey (NHANES) 1999-2004. Am J Kidney Dis 2008;51(4, Suppl 2):S38–45. 10.1053/j.ajkd.2007.12.017 18359407

[22] Go AS , Chertow GM , Fan D , McCulloch CE , Hsu CY . Chronic kidney disease and the risks of death, cardiovascular events, and hospitalization. N Engl J Med 2004;351(13):1296–305. 10.1056/NEJMoa041031 15385656

[23] Martorell R , Khan LK , Hughes ML , Grummer-Strawn LM . Obesity in women from developing countries. Eur J Clin Nutr 2000;54(3):247–52. 10.1038/sj.ejcn.1600931 10713748

[24] Amoah AGB . Sociodemographic variations in obesity among Ghanaian adults. Public Health Nutr 2003;6(8):751–7. 10.1079/PHN2003506 14641945

[25] Finucane MM , Stevens GA , Cowan MJ , Danaei G , Lin JK , Paciorek CJ , National, regional, and global trends in body-mass index since 1980: systematic analysis of health examination surveys and epidemiological studies with 960 country-years and 9.1 million participants. Lancet 2011;377(9765):557–67. 10.1016/S0140-6736(10)62037-5 21295846 PMC4472365

[26] Danaei G , Finucane MM , Lu Y , Singh GM , Cowan MJ , Paciorek CJ , National, regional, and global trends in fasting plasma glucose and diabetes prevalence since 1980: systematic analysis of health examination surveys and epidemiological studies with 370 country-years and 2.7 million participants. Lancet 2011;378(9785):31–40. 10.1016/S0140-6736(11)60679-X 21705069

[27] Mbanya JCN , Motala AA , Sobngwi E , Assah FK , Enoru ST . Diabetes in sub-Saharan Africa. Lancet 2010;375(9733):2254–66. 10.1016/S0140-6736(10)60550-8 20609971

[28] Collins AJ , Foley RN , Herzog C , Chavers B , Gilbertson D , Ishani A , United States renal data system 2008 annual data report. Am J Kidney Dis 2009;53(1, Suppl):S1–374. 10.1053/j.ajkd.2008.10.005 19111206

[29] Barquera S , Campos-Nonato I , Hernandez-Barrera L , Villalpando S , Rodríguez-Gilabert C , Durazo-Arvizú R , Hypertension in Mexican adults: results from the National Health and Nutrition Survey 2006. Salud Publica Mex 2010;52(Suppl 1):S63–71. 10.1590/S0036-36342010000700010 20585731

[30] Misra A , Vikram NK , Gupta R , Pandey RM , Wasir JS , Gupta VP . Waist circumference cutoff points and action levels for Asian Indians for identification of abdominal obesity. Int J Obes (Lond) 2006;30(1):106–11. 10.1038/sj.ijo.0803111 16189502

[31] Gansevoort RT , Verhave JC , Hillege HL , Burgerhof JG , Bakker SJ , de Zeeuw D , PREVEND Study Group. The validity of screening based on spot morning urine samples to detect subjects with microalbuminuria in the general population. Kidney Int Suppl 2005;(94):S28–35. 10.1111/j.1523-1755.2005.09408.x 15752236

[32] Stierle F , Kaddar M , Tchicaya A , Schmidt-Ehry B . Indigence and access to health care in sub-Saharan Africa. Int J Health Plann Manage 1999;14(2):81–105. 10.1002/(SICI)1099-1751(199904/06)14:2<81::AID-HPM543>3.0.CO;2-P 10538937

[33] Epping-Jordan JE , Pruitt SD , Bengoa R , Wagner EH . Improving the quality of health care for chronic conditions. Qual Saf Health Care 2004;13(4):299–305. 10.1136/qshc.2004.010744 15289634 PMC1743863

